# Comparative Analysis of RNA Virome Composition in Rabbits and Associated Ectoparasites

**DOI:** 10.1128/JVI.02119-19

**Published:** 2020-05-18

**Authors:** Jackie E. Mahar, Mang Shi, Robyn N. Hall, Tanja Strive, Edward C. Holmes

**Affiliations:** a Marie Bashir Institute for Infectious Disease and Biosecurity, School of Life and Environmental Sciences and School of Medical Sciences, The University of Sydney, Sydney, NSW, Australia; b Health and Biosecurity, Commonwealth Scientific and Industrial Research Organisation Black Mountain, Canberra, ACT, Australia; c Centre for Invasive Species Solutions, University of Canberra, Bruce, ACT, Australia; University of Texas Southwestern Medical Center

**Keywords:** virus, ectoparasites, transmission, calicivirus, meta-transcriptomics, evolution

## Abstract

Ectoparasites play an important role in virus transmission among vertebrates. Little, however, is known about the nature of those viruses that pass between invertebrates and vertebrates. In Australia, flies and fleas support the mechanical transmission of two viral biological controls against wild rabbits—rabbit hemorrhagic disease virus (RHDV) and myxoma virus. We compared virome compositions in rabbits and these ectoparasites, sequencing total RNA from multiple tissues and gut contents of wild rabbits, fleas collected from these rabbits, and flies trapped sympatrically. Meta-transcriptomic analyses identified 50 novel viruses from multiple RNA virus families. Rabbits and their ectoparasites were characterized by markedly different viromes, with virus abundance greatest in flies. Although viral contigs from six virus families/groups were found in both rabbits and ectoparasites, they clustered in distinct host-dependent lineages. A novel calicivirus and a picornavirus detected in rabbit cecal content were vertebrate specific; the newly detected calicivirus was distinct from known rabbit caliciviruses, while the picornavirus clustered with sapeloviruses. Several picobirnaviruses were also identified that fell in diverse phylogenetic positions, compatible with the idea that they are associated with bacteria. Further comparative analysis revealed that the remaining viruses found in rabbits, and all those from ectoparasites, were likely associated with invertebrates, plants, and coinfecting endosymbionts. While no full genomes of vertebrate-associated viruses were detected in ectoparasites, small numbers of reads from rabbit astrovirus, RHDV, and other lagoviruses were present in flies. This supports a role for flies in the mechanical transmission of RHDV, while their involvement in astrovirus transmission merits additional exploration.

**IMPORTANCE** Ectoparasites play an important role in the transmission of many vertebrate-infecting viruses, including Zika and dengue viruses. Although it is becoming increasingly clear that invertebrate species harbor substantial virus diversity, it is unclear how many of the viruses carried by invertebrates have the potential to infect vertebrate species. We used the European rabbit (Oryctolagus cuniculus) as a model species to compare virome compositions in a vertebrate host and known associated ectoparasite mechanical vectors, in this case, fleas and blowflies. In particular, we aimed to infer the extent of viral transfer between these distinct types of host. Our analysis revealed that despite extensive viral diversity in both rabbits and associated ectoparasites, and the close interaction of these vertebrate and invertebrate species, biological viral transmission from ectoparasites to vertebrate species is rare. We did, however, find evidence to support the idea of a role of blowflies in transmitting viruses without active replication in the insect.

## Abstract (precis)

Ectoparasites play an important role in the transmission of many vertebrate-infecting viruses, including Zika and dengue viruses. Although it is becoming increasingly clear that invertebrate species harbor substantial virus diversity, it is unclear how many of the viruses carried by invertebrates have the potential to infect vertebrate species. We used the European rabbit (Oryctolagus cuniculus) as a model species to compare virome compositions in a vertebrate host and known associated ectoparasite mechanical vectors, in this case, fleas and blowflies. In particular, we aimed to infer the extent of viral transfer between these distinct types of host. Our analysis revealed that despite extensive viral diversity in both rabbits and associated ectoparasites, and the close interaction of these vertebrate and invertebrate species, biological viral transmission from ectoparasites to vertebrate species is rare. We did, however, find evidence to support the idea of a role of blowflies in transmitting viruses without active replication in the insect.

## INTRODUCTION

Ectoparasites act as vectors for many notable viral pathogens of vertebrates (1–3). Transmission can occur “biologically,” with active virus replication in the ectoparasite, or “mechanically,” without ectoparasite replication (2, 4–6). Both mechanisms enable viruses to spread across spatial or ecological barriers that might inhibit direct transmission (7). Ectoparasites are predominantly arthropods, including such animals as lice and fleas, as well as intermittent ectoparasites such as mosquitos, ticks, and blowflies (8).

The European rabbit (Oryctolagus cuniculus) has been profoundly impacted by ectoparasite-mediated viral transmission. As rabbits are a pest species in Australia, two virus biological controls—rabbit hemorrhagic disease virus (RHDV; single-stranded RNA) and myxoma virus (MYXV; double-stranded DNA)—were deliberately introduced to control wild rabbit populations in the 1950s and 1990s, respectively (9). Blowflies (Calliphoridae) and bushflies (Muscidae) are associated with the transmission of RHDV, while two species of rabbit fleas (Spilopsyllus cuniculi and Xenopsylla cunicularis) aid MYXV transmission (and mosquitos are potentially involved in the subsidiary transmission of both viruses) (5, 9–13). As viral replication is not believed to occur in insect tissue, transmission is entirely mechanical. RHDV is ingested by flies during feeding on carcasses and viable virus excreted in fly spots (13), while fleas transmit MYXV through contaminated mouthparts (14).

Despite the importance of the ectoparasite-vector system in virus transmission and evolution, little is known about the composition of virus communities in vertebrates and their associated ectoparasites and particularly how commonly viruses are able to infect both host types. Metagenomic studies of arthropod vector species such as mosquitoes and ticks have revealed an unexpectedly rich diversity of viruses, most of which likely do not infect vertebrates (15, 16). However, it is still unclear what proportion of the viruses present in invertebrates pass to vertebrates and vice versa, although such information is central to understanding the evolution of vector-borne transmission and determining whether some viruses have more liberal host preferences than others.

The advent of bulk RNA sequencing (“meta-transcriptomics”) has revolutionized our perception of viral diversity and host range (17, 18), revealing large numbers of seemingly benign viruses (19). The invertebrate meta-transcriptomic studies undertaken to date have included various species of ectoparasite, such as mosquitos, ticks, and fleas, revealing abundant and complex viromes (15, 16, 20). Here, by comparing the viromes of Australian wild rabbits alongside associated rabbit fleas and sympatric flies, we present a joint study of virome composition in vertebrates and their associated ectoparasites. In particular, we aimed to determine whether and how virome compositions differed between rabbits and the ectoparasites sampled on or near these rabbits and whether some types of virus were common to both host types such that they are involved in either biological or mechanical transmission.

## RESULTS

### Data generated.

RNA sequencing of 10 rabbit sample libraries and 9 invertebrate sample libraries generated a total of 200,290,927 paired-end (PE) reads (40 Gbp) and 260,721,139 PE reads (52 Gbp), respectively, with rabbit library size ranges of 19,127,004 to 21,164,747 PE reads and invertebrate library size ranges of 27,455,130 to 30,538,954 PE reads. Of the reads from the rabbit libraries, 85% did not map to host rRNA, while 96% of invertebrate reads did not map to published host rRNA.

### Genetic identification of unknown arthropods.

The majority of arthropods analyzed in this study were identified to the species level through visual inspection. The remainder were characterized using the transcriptome sequencing (RNA-Seq) data. Fleas were confirmed to be Spilopsyllus cuniculi (rabbit fleas) based on the presence of several highly abundant contigs of Spilopsyllus cuniculi rRNA and EF1a genes and the absence of any other *Spilopsyllus* species genes. A library of unidentified *Chrysomya* species (GUNChsp library) was determined to represent Chrysomya rufifacies or Chrysomya albiceps (these two species are potentially the same) based on EF1a and rRNA gene data. An unknown *Sarcophaga* species was most likely Sarcophaga impatiens based on 28S rRNA identity.

### Fly species trapped.

A wider diversity of flies were trapped at site 1 (Gungahlin), a suburb of Canberra (*n* = 5 species), than at site 2 (Gudgenby) in Namadgi National Park (*n* = 2 species) (Table 1). Species from the genera *Calliphora*, *Chrysomya* (both Calliphoridae), and *Sarcophaga* (Sarcophagidae) were collected from Gungahlin, while species from *Calliphora* and *Musca* (Muscidae) were isolated from Gudgenby. Calliphora augur was the only species trapped at both sites (Table 1). While it was not possible to confirm that the flies trapped and sequenced in this study had interacted with rabbits, there is evidence that *Calliphora*, *Chrysomya*, *Sarcophaga*, and *Musca* species feed on European rabbits in Australia (5, 10).

**TABLE 1:** Rabbit and insect sampling and pooling details

Library	Site^*a*^	Species	Sample type	No. of samples in RNA-Seq pool
Rabbit tissues				
GUN-Bl	1	*Oryctolagus cuniculus*	Blood	20
GUN-Li	1	*Oryctolagus cuniculus*	Liver	20
GUN-Lu	1	*Oryctolagus cuniculus*	Lung	20
GUN-Duo	1	*Oryctolagus cuniculus*	Duodenum	20
GUN-CC	1	*Oryctolagus cuniculus*	Cecal content	20
Gudg-Bl	2	*Oryctolagus cuniculus*	Blood	18
Gudg-Li	2	*Oryctolagus cuniculus*	Liver	18
Gudg-Lu	2	*Oryctolagus cuniculus*	Lung	18
Gudg-Duo	2	*Oryctolagus cuniculus*	Duodenum	18
Gudg-CC	2	*Oryctolagus cuniculus*	Cecal content	18

Arthropods				
GUN-F	1	*Spilopsyllus cuniculi*^*b*^	Entire fleas grouped by rabbit	>70 fleas from 8 rabbits
GUN-ChV	1	*Chrysomya varipes*	Entire fly	10
GUN-Chsp	1	*Chrysomya rufifacies*/*albiceps*^*b*^	Entire fly	10
GUN-CaV	1	*Calliphora vicina*	Entire fly	10
GUN-CaA	1	*Calliphora augur*	Entire fly	10
GUN-Sasp	1	*Sarcophaga impatiens*^*b*^	Entire fly	2
Gudg-F	2	*Spilopsyllus cuniculi*^*b*^	Entire fleas grouped by rabbit	>50 fleas from 7 rabbits
Gudg-CaA	2	*Calliphora augur*	Entire fly	10
Gudg-Un	2	*Musca vetustissima*	Entire fly	2

a Site 1, CSIRO Crace, Gungahlin; Site 2, Gudgenby Valley in Namadgi National Park.

b Species designation based on RNA-Seq data.

### Virus contigs in ectoparasites.

**FIG 1:**
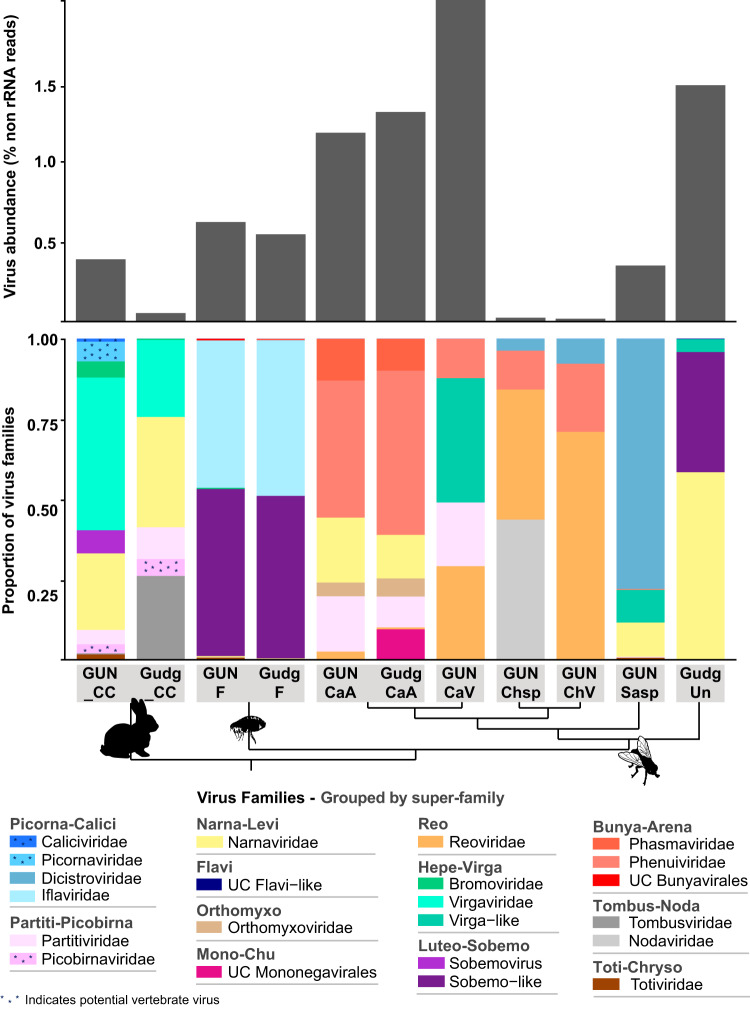
RNA virus abundance and composition in rabbit and invertebrate libraries. The top plot displays the abundance of viral reads (*y* axis) in each library (*x* axis) as a proportion of total non-rRNA reads. The bottom plot shows the viral composition of each library by virus family/group (shaded/grouped by superfamily). Potential vertebrate viruses are indicated by asterisks within the shading. Only RNA viruses (with a RdRp) are shown, and only virus families that had an abundance of at least 0.001% in at least one library are presented. “UC” denotes unclassified viruses. Virus libraries are labeled as follows (collection site—species): GUN_CC, Gungahlin—rabbit (cecal content); Gudg_CC, Gudgenby—rabbit (cecal content); GUNF, Gungahlin—flea; GudgF, Gudgenby—flea; GUNCaA, Gungahlin—Calliphora augur; GudgCaA, Gudgenby—Calliphora augur; GUNChsp, Gungahlin—Chrysomya rufifacies/albiceps; GUNCaV, Gungahlin—Calliphora vicina; GUNChV, Gungahlin—Chrysomya varipes; GUNSasp, Gungahlin—Sarcophaga impatiens; GudgUn, Gudgenby—Musca vetustissima. Note that only the cecal content libraries from rabbits are included in the plots since no viral contigs were found in the other libraries. A cladogram connecting the libraries beneath the *x* axis indicates the relationships between the sampled hosts in each library, where tips represent host species and nodes represent (from top to bottom) the levels of genus, family, superfamily, order, class, and kingdom.

A large number of RNA viral contigs were assembled from the flea and fly libraries. Of the invertebrate species, Calliphora vicina had the highest virus abundance (as a proportion of non-rRNA reads), with almost 2% of non-rRNA reads of viral origin, while *Chrysomya* species had viral abundances of only 0.012% to 0.018% (Fig. 1). Each ectoparasite species had virus contigs from between 4 and 11 different RNA virus families/groups. While viruses from several different families were detected in fleas (8 to 10 families), viruses in both flea libraries largely belonged to the *Iflaviridae* and Sobemo-like viruses (Fig. 1). Of the fly species, both Calliphora augur libraries harbored the highest number of virus families (8 to 11 families), and the *Chrysomya* and *Musca* libraries were the least diverse, with 4 to 5 viral families per library. Although only fleas and Calliphora augur were sampled from both sites, the viral diversity of these two species at each site suggests that viral composition was associated with host species rather than collection location (Fig. 1). The fly results also suggest that there was a trend in viral composition at the genus level (GUNCaA, GudgCaA, and GUNCaV are genus *Calliphora*, while GUNChsp and GUNChV are *Chrysomya*), with decreasing similarity in viral composition at the family level and beyond (Fig. 1).

**FIG 2:**
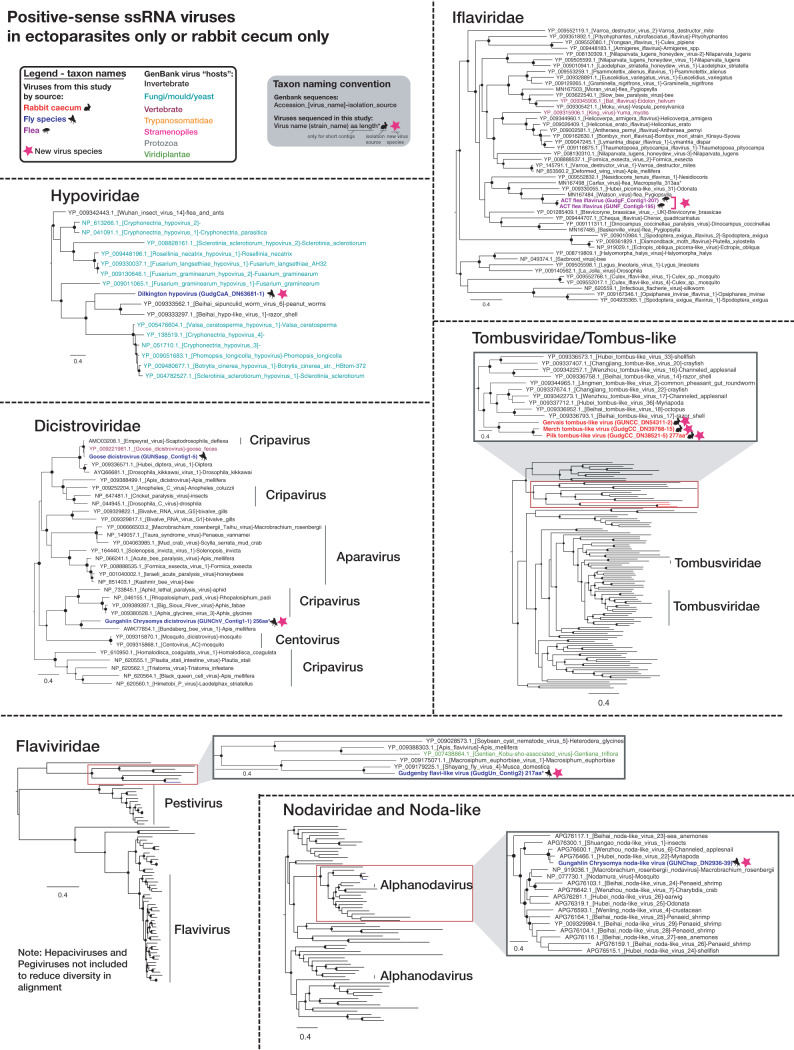
Maximum likelihood (ML) phylogenies of the RdRp of likely nonvertebrate single-stranded positive-sense RNA viruses in ectoparasites only or in the rabbit cecum only. The taxon name (and branches in minimized trees) for sequences obtained in this study are bolded and colored red (rabbit cecal content), blue (flies), or purple (fleas), based on the animal from which they were obtained, with relevant animal symbols adjacent to the names. Viruses that likely constitute a new viral species are indicated by a pink star symbol adjacent to taxon names, and a proposed virus species name is given as the taxon name (with strain name in parentheses). For GenBank sequences, taxon names are colored by the apparent host group from which virus or viral sequence was reportedly isolated as follows: black, invertebrate; teal, fungi/mold/yeast; maroon, vertebrates; orange, Trypanosomatidae; pink, Stramenopiles (microalgae [diatom]/Oomycetes); gray, other protozoa (Coccidia, *Trichomonas*, *Giardia*). SH-like branch support values are represented by circles at the nodes if >0.7 and are sized according to values where the largest circles represent an SH-like support value of 1. For sequences that are less than 80% of the alignment length, the sequence length in amino acids (aa) and an asterisk are included in the taxon name. ssRNA, single-stranded RNA.

**FIG 3:**
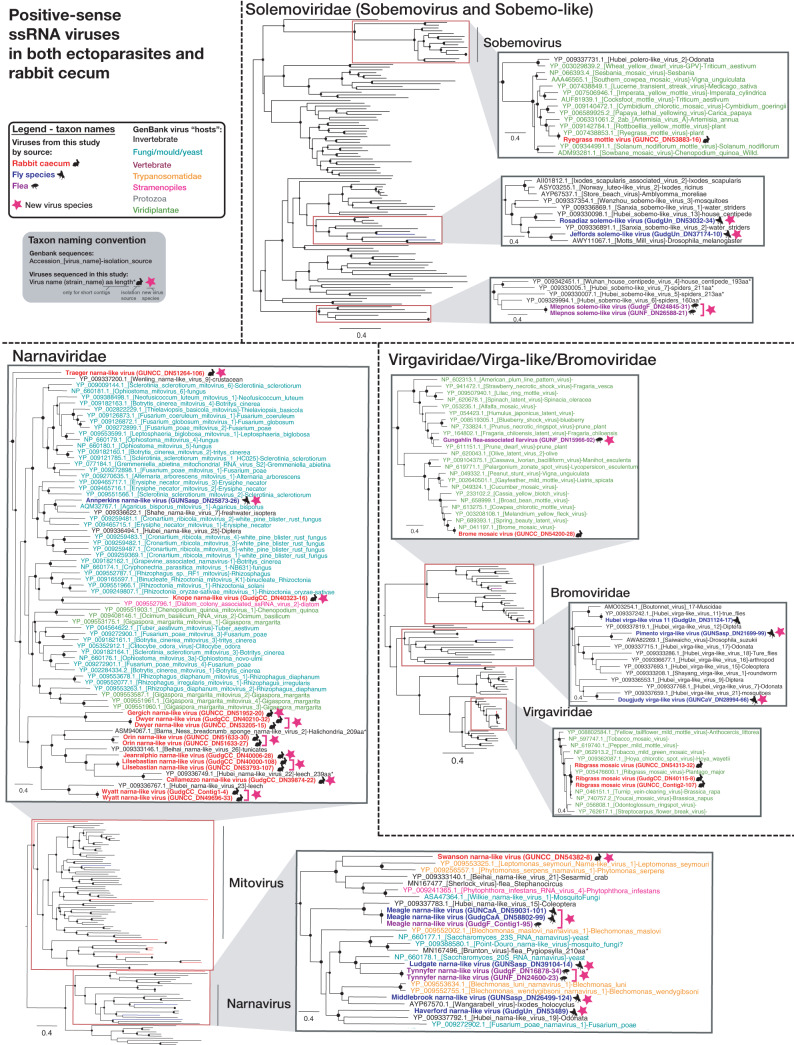
ML phylogenies of the RdRp of likely nonvertebrate single-stranded positive-sense RNA viruses present in both ectoparasites and the rabbit cecum. Figure legend follows Fig. 2.

**FIG 4:**
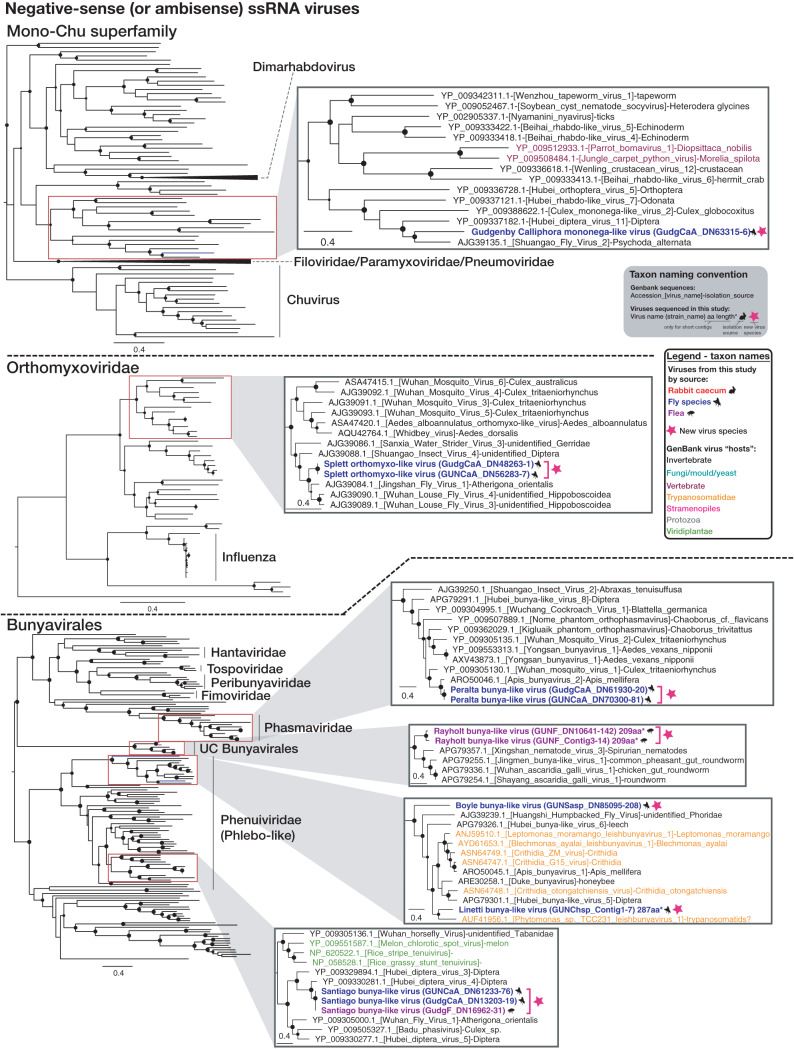
ML phylogenies of the RdRp of likely nonvertebrate negative-sense (or ambisense) RNA viruses. Figure legend follows Fig. 2.

**FIG 5:**
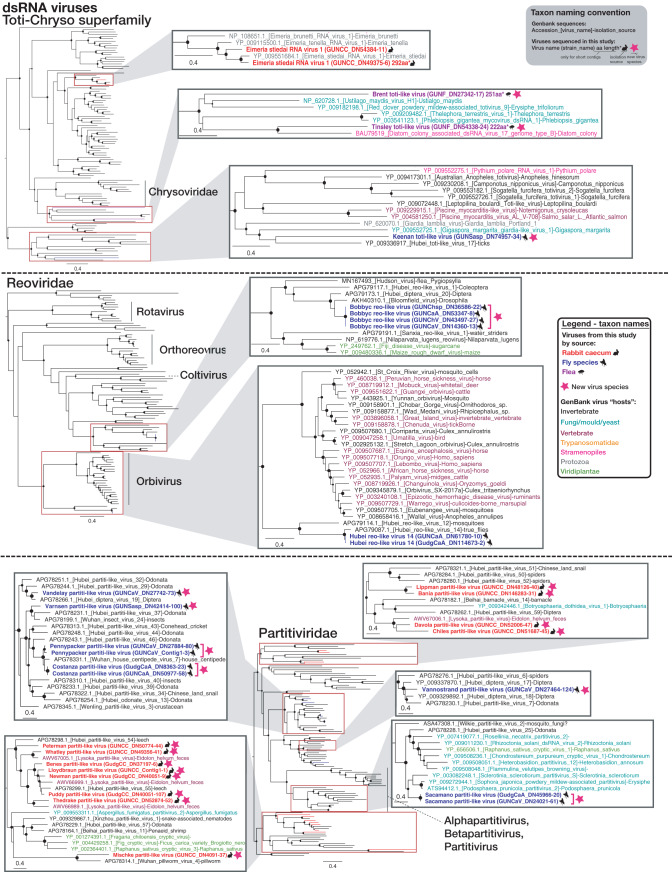
ML phylogenies of the RdRp of likely nonvertebrate double-stranded RNA viruses. Figure legend follows Fig. 2. dsRNA, double-stranded RNA.

To establish the diversity and potential hosts of newly defined viruses, family-level (and, in some cases, superfamily-level) phylogenetic trees were estimated using the virus RNA-dependent RNA polymerase (RdRp) (Fig. 2, 3, 4, and 5). Although many of the highly diverse phylogenies had poorly resolved topologies, we identified at least 30 diverse viruses that likely constitute new species (indicated with stars in Fig. 2, 3, 4, and 5). Species demarcation criteria differ between virus families and even between different genera of the same family, and every attempt was therefore made to define likely new species in accordance with the relevant criteria as defined by the International Committee on Taxonomy of Viruses (ICTV) (where sequence-based criteria exist). At a minimum, all newly defined species shared less than 90% amino acid identity with their closest relative in the RdRp, but most were vastly more divergent. The majority of viruses found in invertebrate species clustered with invertebrate-associated viruses in the *Dicistroviridae*, *Iflaviridae*, *Nodaviridae*, Flavi-like, *Solemoviridae*/Sobemo-like, Virga-like, *Orthomyxoviridae*, *Mononegavirales*, *Reoviridae*, *Phasmaviridae* (*Bunyavirales*), and unclassified *Bunyavirales* groups. Additionally, many of the viruses found in insects, particularly fleas, were potentially viruses of fungi, protozoa, or algae, being present in the *Hypoviridae*, *Narnaviridae*, *Partitiviridae* groups, the *Totiviridae-Chrysoviridae* group, and certain *Phenuiviridae* (*Bunyavirales*). The *Bromoviridae* virus identified in Gungahlin fleas clusters firmly among plant viruses, and with an abundance of only 0.002%, it likely represents a plant virus incidentally carried by one or more fleas in the library rather than a virus that replicates in fleas. Indeed, care must be taken in assigning viruses to hosts on the basis of metagenomic data alone. The *Iflaviridae* flea viruses found in this study clustered most closely with Watson virus (Fig. 2), detected in fleas (*Pygiopsylla*) from Australian marsupials (sharing 68.6% amino acid identity in the RdRp).

### Virus contigs in rabbits.

No viral contigs could be assembled from the rabbit liver, duodenum, or lung libraries. A small number of viral contigs were found in the Gudgenby blood library, but these represented potential contaminants since (i) the rabbits from Gudgenby had been shot, occasionally resulting in perforation of the cecum, which would contaminate blood in the body cavity; (ii) all viruses detected in the blood were also detected in the cecum (including plant viruses unlikely to be in blood); and (iii) no viruses were found in the blood of rabbits from the Gungahlin site where there was no body cavity contamination.

In contrast, the cecal content for rabbits from both sites contained many viruses, with 8 and 11 RNA viral families detected in the Gudgenby and Gungahlin rabbits, respectively (Fig. 1), including over 25 likely new viral species. The viral composition of the rabbit cecal content was less consistent between the two sites than for the invertebrates sampled. This may have been a consequence of sampling only small sections of cecal content but could also reflect differences in diet at each site (predominantly introduced pastures at Gungahlin versus more subalpine native grassland plants at Gudgenby). Regardless, *Narnaviridae* and *Virgaviridae* were both highly abundant in the cecal content of rabbits from both locations, while *Tombusviridae* was a major component of the cecal virome of Gudgenby rabbits (Fig. 1). These three virus families, which made up more than 70% of the total viral abundance in rabbit cecal content at each site, likely represent viruses of the rabbit diet (plants) and commensal/parasitic organisms such as fungi and protists.

**FIG 6:**
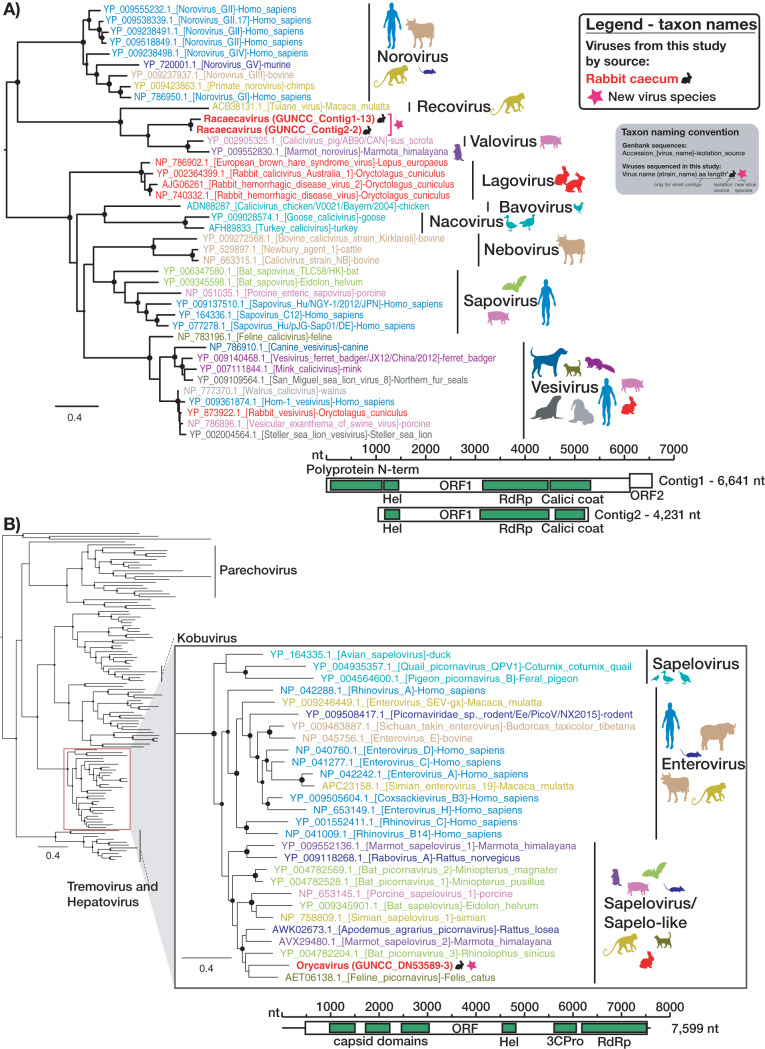
Phylogenetic analysis of the RdRp of vertebrate-specific viruses found in rabbit cecum. ML trees of the RdRp region of (A) the novel rabbit calicivirus—*Racaecavirus*—and (B) the novel rabbit picornavirus—*Orycavirus*—together with representative reference sequences for these virus families are shown. Taxon names of the viruses discovered in this study are bolded with a black rabbit symbol adjacent. A pink star symbol adjacent to taxon names indicates a novel virus species, and the proposed virus species name is given as the taxon name (with strain name in parentheses). GenBank accession numbers are included in the taxon name, and these names are color coded according to host as specified by colored symbols to the right of each tree. Clade labeling indicates specific genera. SH-like branch support values greater than 0.7 are indicated by circles at the nodes, which are sized according to degree of support (an SH-like support value of 1 has the largest size). Trees were midpoint rooted for clarity. The genome structure and length of the isolated contigs are shown below each tree, with open boxes representing ORFs and green boxes indicating conserved protein domains as follows: Polyprotein N-term, N-terminal region of the polyprotein; Hel, helicase; RdRp, RNA-dependent RNA polymerase; Calici coat, calicivirus capsid/coat protein; 3CPro, 3C proteinase.

Although they were less abundant, diverse novel viruses from two vertebrate viral families—the *Caliciviridae* and the *Picornaviridae*—and one potentially vertebrate-associated viral family, the *Picobirnaviridae*, were detected in rabbit cecal content at both sites: all three at Gungahlin and *Picobirnaviridae* at Gudgenby. Two related *Caliciviridae* contigs were assembled, with 77.8% nucleotide identity in the genome, 89.7% amino acid identity in the ORF1 polyprotein, and 90.8% identity in the RdRp protein. They clustered most closely with—although distantly from—a pig calicivirus and a marmot norovirus (Fig. 6), sharing 52% to 54% amino acid identity in the RdRp. Such a divergent phylogenetic position suggests that the calicivirus contigs represent a new viral species that we have termed *Racaecavirus* (Fig. 6). After Sanger sequencing was performed to extend the 3′ end, one of the racaecavirus contigs encompassed a near-complete genome, missing only the 5′ untranslated region (UTR). *Racaecavirus* exhibited a classic form of calicivirus-like genome organization, with two open reading frames (ORF), the first encoding a polyprotein that included RdRp and capsid domains and the second encoding a small protein of unknown function (Fig. 6). Oddly, there appears to have been only one nucleotide in the 3′ UTR of this genome sequence. This was confirmed by 3′ rapid amplification of cDNA ends [using an oligo(dT)-adapter primer] and Sanger sequencing.

Similarly, the entire coding region was obtained for a novel member of the *Picornaviridae*. This contained one large ORF, typical of the *Picornaviridae*, with multiple capsid proteins preceding nonstructural proteins (Fig. 6). The sequence also contained a 5′ (478-nucleotide [nt]) UTR and a 3′ (74-nt) UTR, although it is not clear if these were complete. The novel virus grouped, with strong support, with members of the *Enterovirus* and *Sapelovirus* genera (Fig. 6). The closest sequenced relatives were feline picornavirus, bat picornavirus 3, Apodemus agrarius picornavirus, and marmot sapelovirus 2, which share identity of 61% to 64% with the novel rabbit picornavirus in the RdRp protein (Fig. 6). This level of divergence and phylogenetic position would define this virus as a new species within the genus *Sapelovirus* or within a newly defined sapelovirus-like genus (Fig. 6) (21). Accordingly, we propose the name *Orycavirus*.

Since RNA sequencing was conducted on pools of 18 to 20 samples, specific reverse transcriptase PCRs (RT-PCRs) for the novel racaecavirus and orycavirus identified here were designed to determine their frequency in individual animals. In the Gungahlin cecal content samples, racaecavirus was detected in 4 of 20 samples tested, while orycavirus was detected in 10 of the 20 samples. Despite our finding no racaecavirus or orycavirus contigs in the Gudgenby cecal content library, one sample from this library had a weakly RT-PCR-positive result for both viruses. Subsequent mapping of reads from this library to racaecavirus and orycavirus contigs resulted in 8 and 7 reads mapping to each, respectively. Therefore, these viruses may also occur at a low frequency in Gudgenby.

**FIG 7:**
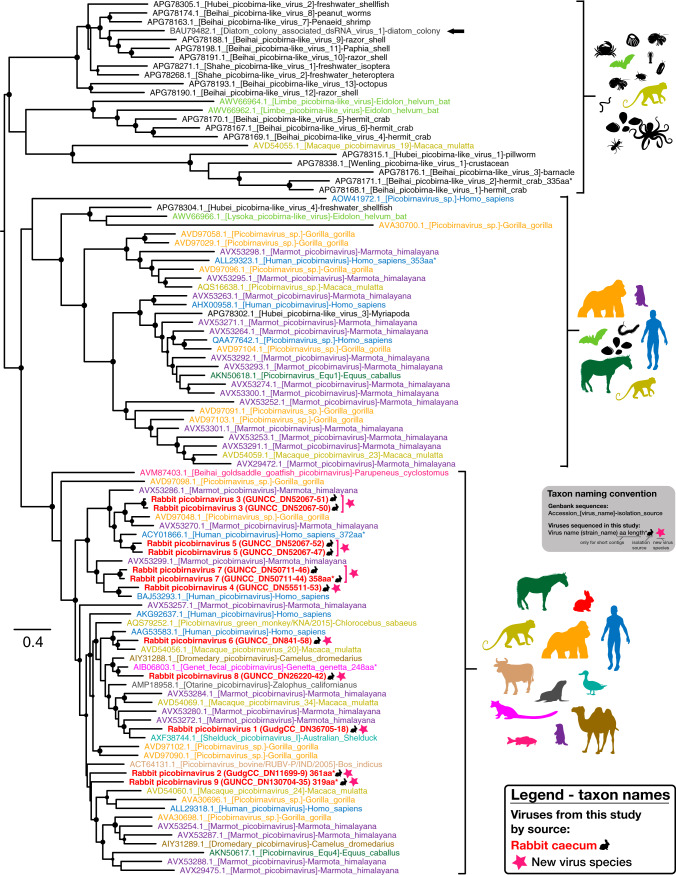
Phylogenetic analysis of the RdRp of novel picobirnaviruses. An ML tree of the RdRp region of novel rabbit picobirnaviruses and representative picobirnaviruses from GenBank is shown. The novel rabbit picobirnavirus taxon names are bolded, colored red, and emphasized with a black rabbit symbol adjacent to the name. A pink star symbol adjacent to taxon names indicates a novel virus species, and the proposed virus species name is given as the taxon name (with strain name in parentheses). The taxon names of GenBank sequences include accession numbers and are colored according to the host taxa from which they were isolated (all invertebrate host taxa are colored black). The host taxa associated with sequences in each clade are indicated with symbols to the right of the clade. The single picobirnavirus sequence isolated from a diatom colony is indicated with an arrow. SH-like branch support values greater than 0.7 are indicated by circles at the nodes, which are sized according to degree of support (an SH-like support value of 1 is maximum size). Trees were midpoint rooted for clarity.

Finally, several picobirnaviruses were identified in rabbit cecal content, all of which clustered strongly in the supposedly vertebrate-specific genogroup 1 clade (Fig. 7). On the basis of the individual species sharing <75% amino acid similarity in the RdRp alignment, these data likely represent nine novel picobirnaviruses (although defined species demarcation criteria for this family are lacking). Consistent with naming conventions, the putative new viruses were named *Rabbit picobirnavirus* 1 to 9. Importantly, these viruses did not form a monophyletic group but were distributed throughout genogroup 1 among picobirnaviruses from different hosts. This pattern is typical of the *Picobirnaviridae*, i.e., showing limited host structure in the RdRp phylogeny (Fig. 7), and is compatible with the idea that these in fact represent bacterium-associated viruses (22). The RdRp segments (corresponding to segment 2) were predicted to have one ORF, consistent with other members of this family. While pairing of segments was difficult, several longer picobirnavirus segments with at least one large ORF, likely encoding the capsid, were identified in both cecal content libraries.

### Virus families present in both insect and rabbit libraries.

**FIG 8:**
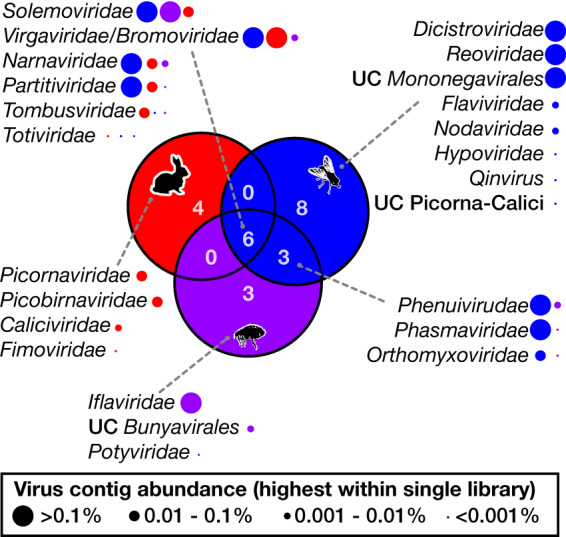
Overlap of RNA viral families in rabbits and ectoparasites. The numbers of viral families/groups for which contigs were assembled from rabbit cecal content libraries (red circle), fly libraries (blue circle), and flea libraries (purple circle) and the level of overlap for each host group are indicated by a Venn diagram. The viral families associated with each segment are listed with gray dotted lines connecting lists to segments of the Venn diagram. The abundance of each viral family for the three groups is indicated by the size of circles next to virus family names. Circles are color coded according to the rabbit, fly, or flea group with which they are associated, and the circle sizes reflect the highest abundance of the relevant virus family within a single library in the rabbit, flea, or fly group. UC, unclassified.

Viral contigs from the *Virgaviridae*/*Bromoviridae*/Virga-like (plant/invertebrate-associated), *Solemoviridae*/Sobemo-like (plant/invertebrate-associated), *Narnaviridae* (fungus/parasite/invertebrate-associated), *Partitiviridae* (plant/invertebrate/fungus/vertebrate feces-associated), *Tombusviridae* (plant/invertebrate-associated), and Toti-Chryso (parasite/invertebrate/fungus-associated) groups were assembled from both the rabbit cecal content and the ectoparasites (Fig. 1 and 8). As noted above, the viruses from these families assembled from rabbit cecal content were unlikely to be actively replicating in rabbits. In addition, assembly of viruses of the same family from both arthropods and rabbits showed that they did not cluster together (Fig. 3 and 5).

To further investigate the viral overlap between rabbits and ectoparasites, reads from ectoparasite libraries were mapped to the viral contigs from rabbit cecal material. A total of 58 viral reads mapped to rabbit virus contigs, all associated with the viral groups described above, and hence likely mapping to conserved regions. Taken together, these results show that no abundant viral species were shared between host and ectoparasites in this sample.

### Low-abundance vertebrate-associated viruses in ectoparasite libraries.

**FIG 9:**
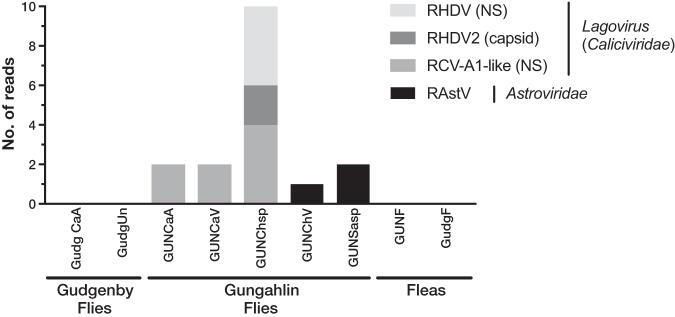
Vertebrate-specific virus reads detected in ectoparasite libraries. The number of reads from vertebrate viruses (*y* axis) detected in ectoparasite libraries (*x* axis) is presented as a stacked bar plot. Vertebrate virus reads that were detected include rabbit astrovirus (RAstV), and RHDV-like viruses that include recombinant variants of RHDV, and the related RHDV2 and RCV-A1. In the legend, “NS” and capsid in parentheses indicate reads mapping to nonstructural genes or the capsid gene, respectively. Ectoparasite libraries are labeled as follows (location—species): GUNF, Gungahlin—flea; GudgF, Gudgenby—flea; GUNCaA, Gungahlin—Calliphora augur; GudgCaA, Gudgenby—Calliphora augur; GUNChsp, Gungahlin—Chrysomya rufifacies/albiceps; GUNCaV, Gungahlin—Calliphora vicina; GUNChV, Gungahlin—Chrysomya varipes; GUNSasp, Gungahlin—Sarcophaga impatiens; GudgUn, Gudgenby—Musca vetustissima.

If the ectoparasites studied here were involved in mechanical transmission, the viruses might not be sufficiently abundant to be assembled into contigs. Therefore, to detect vertebrate viruses at low abundance, we subjected individual reads from the flea and fly libraries to BLASTn and BLASTx analyses. In several fly libraries, small numbers of reads were detected for two known rabbit-specific viruses (Fig. 9): lagoviruses (RHDV and related viruses) of the *Caliciviridae* family and rabbit astroviruses. The lagovirus reads detected included RHDV, rabbit hemorrhagic disease virus 2 (RHDV2), and the benign rabbit calicivirus Australia-1 (RCV-A1). Because of recombination between RHDV, RHDV2, and RCV-A1 (23), classification of these viruses based on small numbers of reads is difficult. However, the presence of reads mapping to the nonstructural gene segments of RHDV and the RCV-A1-like viruses, as well as the structural gene segments of RHDV2, suggests the presence of at least two RHDV-like viruses in these fly libraries—a recombinant RHDV/RHDV2 and recombinant RCV-A1-like/RHDV2. Equivalent read BLAST analyses were conducted on rabbit libraries, and two reads from RHDV2 recombinants were found in each of the Gudgenby liver, Gudgenby lung, and Gungahlin blood libraries. Since they were at very low abundance, these viral reads may represent the early time period of infection or a cleared infection. To provide context for “low abundance” based on BLAST read results, the orycavirus (likely vertebrate) read count was 3,273, while brome mosaic virus, which is likely a plant virus replicated within the gut plant material, had 9,702 reads (note that since orycavirus is genetically divergent, many of the reads are unlikely to return a BLAST result, artificially lowering its abundance). No vertebrate-specific virus reads were detected in the flea libraries or Gudgenby fly libraries. Due to the difficulty in confirming the legitimacy of viral reads, only those that had a virus result for both BLASTx and BLASTn analyses were included. Since diverse viral reads are unlikely to be detected in a BLASTn analysis, this method would have necessarily led to conservative estimates.

Importantly, these viral reads were unlikely to represent contaminants from other libraries since they were not highly abundant in any library and were not present in every library. However, since some viruses were represented by as little as a single read per library, we confirmed the presence of RHDV-like viruses in invertebrates by RT-PCR (23). Notably, several individual flies from all three libraries with RHDV-like reads were positive by RT-PCR, despite each library having only 2 to 10 RHDV-like reads. In addition, bone marrow from rabbit carcasses collected during the same time and at the same location as those of fly trapping at the Gungahlin site were also positive for RHDV2 recombinants by RT-PCR. This, as well as the presence of lagoviruses in rabbits and flies in the wider region at that time (10), suggests that pathogenic lagoviruses were circulating at the time of sampling and that the low numbers of reads in the fly libraries were bona fide. In contrast, no legitimate mapping occurred when ectoparasite reads were mapped to a MYXV reference genome (GenBank accession no. NC_001132.2). This is consistent with the absence of visible clinical signs of myxomatosis in the sampled rabbits. In addition, no viruses with known pathogenic potential in humans were detected in fleas or flies.

## DISCUSSION

Ectoparasites are rich in virus diversity (17, 24), and many ectoparasite species are involved in the transmission of viruses that affect vertebrates (2, 13). A key question is what proportion of the viruses detected in ectoparasites are potentially transmissible to their vertebrate hosts and vice versa, through either the biological or mechanical transmission route. These could include benign viruses which are transmitted from ectoparasites to vertebrates but which have not been reported due to a lack of detectable disease. Similarly, it is important to determine whether some viruses have a greater propensity for mechanical transmission or a greater capacity to productively infect both vertebrates and invertebrates.

To better understand the extent of viral overlap of vertebrates and associated ectoparasites and the types of viruses present in both, we compared the viromes of apparently healthy Australian wild rabbits to those of associated fleas and sympatric flies. These ectoparasites are known to be involved in the transmission of rabbit viruses (12, 13). No viral contigs were assembled from the lung, liver, duodenum, or blood of rabbits, suggesting the absence of acute or chronic systemic infection in the wild rabbits sampled for this study. In contrast, considerable viral diversity was detected in the cecal content. This likely reflects the role that this organ plays in the digestion of plant matter, such that it is rich in bacteria, other microorganisms, and semidigested plant material (25, 26). On the basis of phylogenetic position, most viruses identified in the cecal content were likely associated with the rabbit diet or other commensal microorganisms, such as fungi and protozoa (Fig. 2, 3, and 5). This includes viruses from the *Narnaviridae*, *Solemoviridae*, *Bromoviridae*, *Virgaviridae*, *Partitiviridae*, *Tombusviridae*, and Toti-Chryso. Those in the *Narnavirdae*, *Partitiviridae*, and *Tombusviridae* likely constitute new viral species (Fig. 2, 3, and 5). Notably, although the members of the *Tombusviridae* family have traditionally been identified as associated with plants, recent studies have found many tombus-like viruses in invertebrates (17) and these group with the cecal content viruses determined here. Hence, these tombus-like viruses may in fact infect commensal or parasitic microorganisms such as protists or fungi, or the rabbits may have been incidentally eating invertebrates. To our knowledge, equivalent viral meta-transcriptomics analyses of cecal content have not been reported, although an abundance of plant and microorganism-associated viruses is consistent with the fecal viromes of other herbivores (27–29). Importantly, we identified in rabbits diverse novel viruses—*Racaecavirus* and *Orycavirus*—that cluster with other vertebrate-associated viruses (in the *Caliciviridae* and the *Picornaviridae*, respectively) (Fig. 6), suggesting that the most likely hosts are the rabbits from which they were sampled. In addition, several novel picobirnaviruses were detected (Fig. 7), although their true host is uncertain. Overall, the abundance of the potential vertebrate viruses detected in rabbits was relatively low, with a calicivirus level of 0.003%, a picornavirus level of 0.025%, and picobirnavirus levels of 0.002% to 0.011%, although benign rabbit viruses have been previously shown to be present at low titer (30, 31). As these viruses were isolated from cecal content, we would not expect to have sampled a high proportion of rabbit cells and viruses replicating in these cells.

Members of the *Caliciviridae* and *Picornaviridae* are frequently detected in vertebrates (19, 21), with many cases of confirmed host association (32–35). The *Caliciviridae* can be associated with serious illnesses, such as gastroenteritis in humans (36) and hemorrhagic disease in rabbits (37), while the members of the *Picornaviridae* are a diverse group of viruses associated with various diseases in humans and animals (21). Although there are two existing genera that include rabbit caliciviruses, rabbit *Vesivirus* and *Lagovirus*, the novel rabbit calicivirus identified here, *Racaecavirus*, was most closely related to a pig calicivirus (*St-Valerian swine virus*) and *Marmot norovirus* (Fig. 6), both sampled from the gut of healthy animals (38, 39). *St-Valerian swine virus* is the only species within the newly classified genus *Valovirus*, and the virus identified here (together with *Marmot norovirus*) likely belongs to that genus (38). The novel rabbit picornavirus that we identified in cecal content, *Orycavirus*, was phylogenetically distinct from other rabbit picornaviruses, clustering with enteroviruses and sapeloviruses/sapelo-like viruses (Fig. 6). Members of the genus *Enterovirus* include important human respiratory pathogens and have been shown to be associated with more-serious symptoms such as acute flaccid myelitis, meningitis, myocarditis, and encephalitis (34). Enteroviruses primarily target the gastrointestinal tract, and most infections are thought to be asymptomatic (34). The genus *Sapelovirus* was initially classified with members from swine, primate, and avian hosts, with an unclear link to pathogenicity (40), although the creation of several new genera may now be appropriate (21). The closest relatives of orycavirus were isolated from feces of apparently healthy cats, bats, and marmots, as well as from rodents with unknown disease status (39, 41, 42). It is notable that the calicivirus and picornavirus detected here clustered with other viruses isolated from the gut content of seemingly healthy vertebrate hosts, tentatively suggestive of cellular tropism specific to the intestinal tract. Additionally, since the sampled rabbits were apparently healthy, the novel caliciviruses and picornaviruses are likely nonpathogenic. Whether these viruses were present in the founder population of rabbits first introduced into Australia or whether they were exotic incursions awaits additional sampling.

Nine novel species of *Picobirnaviridae* were identified in the rabbit cecum. *Picobirnaviridae* have been detected in several vertebrate species, including rabbits (43–45) as well as invertebrates (17) and diatom colonies (46). The picobirnaviruses documented here all clustered with the highly diverse and seemingly vertebrate-associated genogroup 1. The new viruses do not form a monophyletic group by host species (Fig. 7), consistent with other members of this family, and diverse picobirnaviruses are commonly found in a single species (45, 47). Consistent with our detection of *Picobirnaviridae* in cecal content, viruses of this family have commonly been isolated from stool samples or cloacal swabs of vertebrates, either with no apparent symptoms or associated with diarrhea (48–51). Although it has been suggested that these viruses are opportunistic pathogens (44), the absence of a host phylogenetic structure and lack of conclusive detection in solid tissues suggest that vertebrates and invertebrates may not be the true hosts of this virus family. Indeed, based on the presence of conserved prokaryotic ribosomal binding sites, it was recently proposed that prokaryotes are the true hosts of *Picobirnaviridae* (22), which would accord with the lack of taxonomic structure in vertebrate hosts.

A large number of diverse viruses (including >30 novel species) were discovered in fleas collected from rabbits and Calliphoridae, Sarcophagidae, and Muscidae flies trapped sympatrically (Fig. 1 to 5 and 8). Viral composition in ectoparasites varied according to host species (Fig. 1) rather than location, consistent with the pattern seen in Australian mosquitos (16). The majority of highly abundant viruses were invertebrate viruses, with the remainder likely representing viruses of fungi, protozoa, or other commensal microbes (Fig. 2 to 5). Several viral families/groups identified in rabbit flea libraries were also found in fleas collected from Australian marsupials or rats, including *Solemoviridae*, *Iflaviridae*, *Narnaviridae*, *Phenuiviridae*, and *Totiviridae* (20). Generally, viruses from rabbit fleas did not cluster with viruses from other flea species (Fig. 2 to 5), with the exception of the *Iflaviridae* flea viruses, which were most closely related to Watson virus, a virus of *Pygiopsylla* fleas collected from an Australian marsupial (20). Viruses from six different viral groups/families were identified in both ectoparasites and rabbits (Fig. 8), although the ectoparasite viruses were phylogenetically distinct from those found in rabbit cecal samples (Fig. 3 and 5). There is therefore no evidence of viral transfer between these ectoparasites and rabbits. Indeed, no highly abundant vertebrate viruses or known arboviruses were found in flies or fleas, suggesting that potential arboviruses are not frequently circulating in these arthropods. However, a larger sample size is needed to clarify the role of these ectoparasites in biological transmission.

Carrion/bush flies and fleas have been implicated in the mechanical transmission of RHDV and MYXV in rabbits (5, 10, 12, 13). In these cases, no viral replication takes place in the ectoparasite, such that viral abundance would be very low and viral contigs might not be assembled. To detect viruses potentially associated with mechanical transmission, we also explored the low-abundance viral reads from the invertebrate libraries (i.e., reads which were not assembled into contigs). This revealed evidence of the presence of RHDV and related lagoviruses (*Caliciviridae*) in three Calliphoridae fly species (Fig. 9)—a family of flies associated with RHDV transmission (5, 10, 13). Since the introduction of RHDV into Australia in 1995, several related viruses have been detected, including recombinants of RHDV and RHDV2 or of benign RCV-A1 viruses and RHDV2 (23, 52, 53). At least two RHDV2 variants were detected in fly reads (RHDV/RHDV2 and RCV-A1-like/RHDV2), both known to be circulating at that time (10, 23, 54), and were confirmed by RT-PCR. A small number of RHDV2 reads were also identified in rabbit libraries, and incidentally, RHDV2 was detected by RT-PCR in dead rabbits found synchronously at the study site. Since RHDV infection is generally acute and susceptible rabbits die rapidly (9), RHDV-like reads were likely detected from recovering animals, in which RHDV RNA is detectable for at least 15 weeks postinfection (55). These results demonstrate that mechanically transmitted viruses can be detected concurrently in the vertebrate host and ectoparasite using a metagenomic approach, even in the case of highly virulent viruses not known to cause persistent infections. Interestingly, rabbit astrovirus was also detected in Sarcophaga impatiens and Chrysomya varipes, although no reads were detected in rabbit material. This virus has been found to be associated with enteric disease in rabbits but may be detected in the gut in the absence of symptoms (56). The detection of rabbit astrovirus in flies is of interest as it suggests that astrovirus may be present in Australian wild rabbit populations and must be shed at high titers if it was acquired from feces. However, as we did not detect any reads in healthy rabbits, more work is clearly needed to establish whether rabbit astroviruses can be transmitted by arthropods.

No viruses known to infect humans or, indeed, any other vertebrates besides leporids were detected in the sampled flies. These fly species are attracted to carrion and feces, a factor that would promote the mechanical transmission of excreted viruses or of those present in carcasses (57). Due to their excessive numbers, rabbit carcasses and feces are not uncommon in rabbit-infested areas (such as the sampling locations), whereas human remains and feces are usually rarer and less accessible. However, we might have expected to find more viruses of livestock (Gungahlin) and native vertebrate species (both sites), which are abundant in the sampling locations. Hence, vertebrate-associated viral mechanical transmission by fly species may be uncommon, and factors such as high prevalence and high virus load in carcasses or feces—as seen for RHDV-like viruses—may therefore be necessary for mechanical transmission (54, 58). In contrast to the results seen with flies, no vertebrate virus reads—including MYXV—were detected in fleas, although their behavior of feeding on vertebrate blood rather than carcasses and feces may limit opportunities for mechanical transmission to periods of acute systemic or viremic infections. As such, ectoparasite behavior and host preference, alongside viral pathogenesis and prevalence, are likely important for mechanical transmission.

In sum, while rabbits and ectoparasites carry viruses from some of the same viral families, viruses from ectoparasites are phylogenetically distinct from viruses found in rabbit cecal content, suggesting that invertebrate viruses rarely establish productive replication cycles in vertebrates. Importantly, however, flies carried a very low abundance of vertebrate viruses with pathogenic capacity, including RHDV, in rabbits, for which fly-mediated mechanical transmission has been demonstrated.

## MATERIALS AND METHODS

### Tissue sampling.

Sampling was performed at two sites within the Australian Capital Territory (ACT), Australia. Site 1 was at the Commonwealth Scientific and Industrial Research Organisation (CSIRO) Crace (−35.22, 149.12), Gungahlin (GUN), a suburb of Canberra, while site 2 was at Gudgenby Valley (−35.74, 148.98) in Namadgi National Park (Gudg). At Gungahlin, rabbits were trapped in carrot-baited cages and killed by cervical dislocation. Trapping occurred over 3 to 5 consecutive nights for two separate weeks of the 2016/2017 Southern Hemisphere summer (18 to 22 December 2016 and 8 to 11 January 2017). A total of 20 rabbits were sampled (60% female), with weights ranging between 0.27 kg and 1.95 kg (mean, 0.82 kg). At Gudgenby, rabbits were killed by shooting on 2 February 2017. Eighteen rabbits were collected (39% female), weighing between 0.52 kg and 2.2 kg (mean, 1.49 kg). Blood (in EDTA tubes), lung, liver, duodenum, and cecal content were collected from each rabbit. Where fleas were present on rabbits, they were collected and grouped by rabbit. Tissues and fleas were stored below –80°C immediately after collection. Sampled rabbits displayed no obvious signs of serious pathology.

Commercially available fly traps (Envirosafe) were placed at the same locations in the same weeks as the rabbit sampling. Traps were baited with rabbit tissue/gut content and/or chicken necks, and bait was physically separated from flies to prevent contamination. Fly traps were left out for periods of up to 24 h. To ensure fresh samples, only live flies were taken from traps. Live flies were chilled at 4°C or –20°C for periods of 5 to 10 min to allow initial visual identification of fly species before they were frozen at –80°C. A total of 149 flies representing 5 species were collected from Gungahlin, while 22 flies from 2 species were collected from Gudgenby (Table 1). While the flies were trapped in the same location and at the same time as the sampled European rabbits, it was not possible to ascertain whether the trapped flies had interacted with rabbits.

All work was carried out according to the Australian Code for the Care and Use of Animals for Scientific Purposes with approval from the institutional animal ethics committee (permit CWLA-AEC#16-02).

### RNA extraction.

RNA was extracted separately for each sample from 20 mg of rabbit tissue or bone marrow, 75 μl of rabbit blood, individual whole flies, or groups of at least 5 fleas from individual rabbits. RNA was extracted using a Maxwell 16 LEV simplyRNA tissue kit in combination with a Maxwell nucleic acid extraction robot (Promega, WI, USA), according to manufacturer’s instruction, including DNase treatment.

### Library construction and sequencing.

Rabbit RNA was pooled by tissue type and collection site, with up to 20 individuals per pool, while insect RNA was pooled by species and collection site, with pool sizes ranging from 2 to 10 individuals (Table 1). Where large numbers of flies of the same species were collected, RNAs from a maximum of 10 flies were pooled. Liver RNA required further DNase treatment after pooling, using Invitrogen Turbo DNase (Thermo Fisher Scientific). All pooled RNA was further purified using an RNeasy MinElute cleanup kit (Qiagen, Hilden, Germany) and quantified using a Qubit RNA broad-range assay with Qubit Fluorometer v3.0 (Thermo Fisher Scientific). RNA pools were assessed for quality using an Agilent RNA 6000 Nano kit and an Agilent 2100 Bioanalyzer (Agilent Technologies, CA, USA). Library construction and sequencing were performed at the Australian Genomic Research Facility. Libraries were constructed using the TruSeq total RNA library preparation protocol (Illumina, CA, USA), and rRNA was removed using an Illumina Ribo-Zero gold rRNA removal kit (Epidemiology). Paired-end (100-bp) sequencing of each RNA library was performed on a HiSeq 2500 sequencing platform (Illumina, CA, USA).

### Assembly and genome annotation.

*De novo* assembly of reads into contigs was performed using Trinity (59) following trimming with Trimmomatic (60). The RSEM tool (61) in Trinity was used to calculate the relative abundances of the contigs (expected counts). BLASTn and DIAMOND BLASTx were then used to compare Trinity contigs to the NCBI nucleotide (nt) database (E value cutoff, 1 × 10^−10^) and nonredundant protein (nr) database (E value cutoff, 1 × 10^−5^), respectively. Results were filtered so that only contigs that had a viral hit (excluding endogenous viruses/retroviruses) from each BLAST search were retained.

Equivalent BLAST analyses were performed on individual reads to detect viruses at low abundance, with E value cutoffs of 1 × 10^−4^ for BLASTx and 1 × 10^−10^ for BLASTn. A conservative approach was taken such that only those reads that had a virus result in both the BLASTn and BLASTx analyses were considered legitimate hits. Ectoparasite library read-mapping to specific virus reference sequences or rabbit viral contigs was conducted using Bowtie2 (62).

To quantify the amount of residual host rRNA sequences in each data set, all reads were mapped to host rRNA using Bowtie2 (62). The rabbit host rRNA target index was generated from a complete O. cuniculus 18S rRNA reference sequence obtained from GenBank (accession no. NR_033238) and a near-complete O. cuniculus 28S rRNA sequence obtained from the SILVA high-quality ribosomal database (63) (accession no. GBCA01000314). The arthropod rRNA target index was generated from 18S and 28S GenBank sequences from Spilopsyllus cuniculi and multiple *Chrysomya*, *Calliphora*, *Sarcophaga*, and *Musca* species. The total number of reads that did not map to host rRNA for each library was used as the denominator to calculate the percentage of reads mapped to viral contigs.

The Geneious assembler (64) was used to extend viral contigs where possible. Open reading frames of viral contigs were identified using the online GeneMark heuristic approach to gene prediction (65), while conserved domains were identified using RSP-TBLASTN v2.6.0, a variant of PSI-BLAST (66).

To mitigate the reporting of false positives due to barcode hopping, a virus was presumed to be a contaminant from another library if it met all three of the following criteria: (i) the virus was detected in at least a third of the libraries sequenced on the same sequencing lane, (ii) the read count representing the abundance of the virus was less than 0.1% of that representing the highest count for that virus among the other libraries, and (iii) the virus shared >99% nucleotide sequence identity with a virus from another library. These criteria were defined on the basis of the observations that barcode hopping usually results in contamination of most libraries on a lane, the index hopping rate is usually about 0.01% to 0.1%, and viral sequences from barcode hopping would be genetically identical to a virus from another library (or ∼99% identical if allowing for a 1% sequencing error rate).

### Phylogenetic analyses.

Reference RNA-dependent RNA polymerase (RdRp) amino acid sequences for each virus family were downloaded from NCBI and aligned with viral contigs using MAFFT v7.271 (67). Where necessary, large data sets were condensed to a more manageable size using CD-HIT version 4.8.1 (68). Poorly and ambiguously aligned sites were removed using trimAl v1.2rev59 (69). Alignments were visualized in Geneious (64). Maximum likelihood trees of each alignment were inferred using PhyML (70) employing the LG amino acid substitution model selected by IQTree (71) and using a combination of NNI (Nearest Neighbor Interchange) and SPR (Subtree Pruning and Regrafting) branch-swapping programs. Branch supports were estimated with the Shimodaira-Hasegawa (SH)-like approximate likelihood ratio test (70). The size and length of each alignment are provided in Table 2, and details of the viral contigs included in phylogenies are provided in Table S1 in the supplemental material.

**TABLE 2:** Details of the virus sequence alignments used in the phylogenetic analyses

Virus family/group	RNA virus type	Alignment length^*a*^	No. sequences in alignment
*Caliciviridae*	Positive sense	397	40
*Picornaviridae*	Positive sense	412	114
*Picobirnaviridae*	Positive sense	468	99
*Solemoviridae*	Positive sense	303	109
*Tombusviridae*	Positive sense	356	113
*Iflaviridae*	Positive sense	448	47
*Narnaviridae*	Positive sense	304	107
*Dicistroviridae*	Positive sense	419	32
*Flaviviridae*	Positive sense	301	69
*Virgaviridae/Bromoviridae*	Positive sense	385	75
*Nodaviridae*	Positive sense	403	63
*Hypoviridae*	Positive sense	258	17
*Orthomyxoviridae*	Negative sense	362	46
*Mononegavirales/Chuviridae*	Negative sense	526	135
*Bunyavirales*	Negative or ambisense	475	145
*Totiviridae/Chrysoviridae*	Double stranded	387	103
*Reoviridae*	Double stranded	444	97
*Partitiviridae*	Double stranded	270	109

a Alignment length data refer to the final length of the alignment in amino acids, after trimming with TrimAl.

### Screening PCRs for detection of rabbit calicivirus and picornavirus.

Primer sets were designed to amplify a small region of each of the novel rabbit calicivirus and picornavirus genomes for the detection of these viruses in individual cecal content samples. Calicivirus primer set GRC_F5.6 (5′-TTA CTC AGA GCG ACC AAG TGC-3′, positive sense) and GRC_R5.9 (5′-CCA GTT CTC GCC TGT ATC CAG-3′, negative sense) amplified a 278-bp region, while picornavirus primer set GRP_F6.5 (5′-GAT CTT ATC CCA CCC AAT CGT GA-3′, positive sense) and GRP_R6.9 (5′-ATA GCC TCT TCT CCA TAA CCA AGC-3′, negative sense) amplified a 401-bp region. RT-PCRs were conducted using a Qiagen OneStep Ahead RT-PCR kit according to the manufacturer’s directions with 1 μl of RNA (diluted 1:10 in nuclease-free water) in a 10-μl reaction volume with a 0.25 μM concentration of each primer. PCR conditions included 10 cycles of touchdown PCR, with the annealing temperature decreasing by 0.5°C each cycle from a starting temperature of 60°C and a further 30 cycles with annealing temperature at 55°C. Representative amplicons were subjected to Sanger sequencing to confirm their legitimacy.

### Extension/confirmation of 3′ end of novel calicivirus genome.

First-strand cDNA synthesis was conducted using an Invitrogen SuperScript IV reverse transcriptase system (Thermo Fisher Scientific, MA, USA), with 5 μl of RNA and 0.5 μM GV270 gene-specific primer (72) in a 20-μl reaction volume. PCR was conducted using an Invitrogen Platinum *Taq* Polymerase High Fidelity kit according to the manufacturer’s protocol with specifically designed forward primer GRC_F6.2 (5′-CAG AGA ATG AGC TCA ACC GAC A-3′) and reverse primer GV271 (72). Reaction volumes of 40 μl included 2.5 μl of cDNA template and a 1 μM concentration of each primer. PCR was conducted for 45 cycles, with the annealing temperature starting at 65°C and decreasing by 0.5°C each cycle. The positive amplicon was approximately 500 bp [including poly(A) tail] and was subjected to Sanger sequencing for confirmation.

### Detection of lagoviruses in flies and rabbit carcasses.

RNAs from individual flies and from the bone marrow of rabbit carcasses found near Gungahlin fly traps were screened for the presence of pathogenic lagoviruses using a multiplex RT-PCR method described previously (23).

### Data availability.

All raw data (fastq files) generated for this study are available in the NCBI SRA database under BioProject accession number PRJNA594431 and BioSample accession numbers SAMN13518403 to SAMN13518421. The new virus nucleotide (consensus) sequences presented in phylogenies are available in NCBI/GenBank under accession numbers MT129676 to MT129780.

## Supplementary Material

Supplemental file 1
